# Volunteering and Metabolic Syndrome and Diabetes in Black Adolescents From Low-Income Families

**DOI:** 10.1001/jamanetworkopen.2025.53419

**Published:** 2026-01-12

**Authors:** Edith Chen, Sarah O. Germer, Hee Moon, Johanna Dezil, Robin Hayen, Tianyi Yu

**Affiliations:** 1 Institute for Policy Research, Northwestern University, Evanston, Illinois; 2 Department of Psychology, Northwestern University, Evanston, Illinois; 3 School of Medicine, University of Minnesota, Minneapolis; 4 College of Medicine, University of Illinois, Chicago; 5 Center for Family Research, University of Georgia, Atlanta

## Abstract

**Importance:**

Disparities by race and socioeconomic status in cardiometabolic diseases are prevalent, and social determinants of health (SDOH) are significant contributors to these disparities. However, SDOH research often focuses on negative factors.

**Objective:**

To examine associations of volunteering, a potentially positive SDOH, with metabolic syndrome and diabetes across 2 samples of Black adolescents from lower-income households, and to assess mediators of associations.

**Design, Setting, and Participants:**

This cohort study used data from 2 studies. Study 1 was a cross-sectional study of Black adolescents (ages 14-19 years) in households with less than 2 times the poverty threshold from 2018 to 2022. Study 2 was a 14-year longitudinal study of Black adolescents (ages 11-20 years) from low-income families from 1994 to 2008. Data were analyzed from April 2024 to November 2025.

**Exposure:**

Study 1 examined volunteering as a binary yes/no as well as volunteering frequency; study 2 examined volunteering from ages 12 to 18 years.

**Main Outcomes and Measures:**

Study 1: metabolic syndrome (counts of the number of metabolic syndrome components above clinical cutoffs; composite z scores of each metabolic syndrome component). Study 2: diabetes diagnosis at age 29.

**Results:**

Study 1 included 400 Black adolescents (256 [64.0%] female; mean [SD] age, 16.39 [1.56] years). Study 2 included 979 Black adolescents (570 [58.2%] female; mean [SD] age, 15.87 [1.71] years). In study 1, a higher frequency of volunteering was associated with a lower metabolic syndrome composite score (*b* = −0.06; SE, 0.03; *P* = .03). Purpose in life, but not physical activity or depressive symptoms, statistically mediated associations between volunteering and metabolic syndrome. In study 2, participants who volunteered during adolescence had lower odds of adult diabetes (odds ratio, 0.48; 95% CI, 0.24 to 0.94; *P* = .03).

**Conclusions and Relevance:**

These results suggest that encouraging adolescents to volunteer may represent a novel approach to improving cardiometabolic health earlier in life and in groups that have been historically marginalized. The notion that volunteering might help not only the recipients of volunteering efforts, but also the volunteers themselves, suggests the possibility of creating a 2-way street of benefits through a single activity that could promote health across groups while also strengthening the social fabric across communities.

## Key Points

**Question:**

Is volunteering associated with metabolic syndrome or diabetes among Black adolescents from lower-income households?

**Findings:**

In this cohort study including 1379 Black adolescents from 2 separate studies, those who volunteered, and who volunteered more frequently, had fewer indications of metabolic syndrome; higher purpose in life statistically mediated this association. Also, Black adolescents who volunteered had reduced odds of diabetes in adulthood.

**Meaning:**

These findings suggest that activities intended to benefit others may relate to better cardiometabolic health among volunteers themselves, suggesting that volunteering could be one positive component of the social determinants of health.

## Teaser

This cohort study examines associations of volunteering, a potentially positive social determinant of health, with metabolic syndrome and diabetes across 2 samples of Black adolescents from lower-income households.

## Introduction

Social determinants of health (SDOH) have increasingly been recognized as important to address because of their role in health outcomes.^1,2,3,4^ However, much of this literature has focused on negative factors (eg, racism, poverty, neighborhood conditions at the structural level,^5,6,7^ social isolation, health-compromising behaviors at the individual or social network level^8,9,10,11^). In considering positive factors, one understudied behavior that may be helpful to understand is volunteering (eFigure in Supplement 1).

Volunteering is defined as behaviors intended to benefit others without an expectation of pay.^12^ There are established psychosocial benefits of volunteering,^13,14,15^ and more recently, research has demonstrated volunteering’s associations with physical health as well. One meta-analysis documented a reliable reduction in mortality of approximately 25% associated with volunteering,^16^ with more recent studies confirming these associations.^17,18^ In addition, volunteering has been associated in observational studies with lower levels of hypertension,^19,20,21^ incident cardiovascular disease (CVD),^18,22^ metabolic syndrome,^21^ and inflammation.^23,24^

These studies have focused predominantly on older adults. However, from a prevention perspective, health-relevant behaviors are important to initiate at younger ages to reduce rates of clinical diseases in adulthood.^25,26^ Yet little is known about whether volunteering done earlier in life is associated with health outcomes. Two previous studies found no association between volunteering during the young adult years and metabolic risk or allostatic load measured in adulthood.^27,28^ However, another study demonstrated that random assignment to volunteering during high school resulted in lower levels of obesity, cholesterol, and inflammation in students compared with a control group that did not volunteer.^29^ To our knowledge, no other studies investigated links between volunteering and cardiometabolic health (CMH) in adolescents.

 Additionally, previous studies used samples that included predominantly predominantly White participants and those with higher socioeconomic status (SES). These individuals are more likely to volunteer.^19,28,30^ However, little is known about links between volunteering and CMH among marginalized groups. This is in spite of there being pronounced disparities in, and burdens resulting from, CVD by race and SES.^31,32,33,34^ One study found that volunteering was associated with more favorable CMH in Hispanic adults.^35^ However, another study found associations between volunteering and hypertension among White, but not Black, older adults.^36^ To our knowledge, there are no studies on volunteering and CMH in adolescents from marginalized groups, such as Black individuals with low income.

This study addresses this gap by conducting an investigation of volunteering and CMH using 2 independent samples of Black adolescents from lower-income households. The first study (hereafter, *study 1*) was a cross-sectional study of Black adolescents investigating associations between volunteering and metabolic syndrome. In line with our theoretical model (eFigure in Supplement 1) of pathways between volunteering and health, this study tested explanations for volunteering and health associations, including individual-level cognitive (greater purpose in life^37,38^), emotional (fewer depressive symptoms^13,14^), and behavioral (greater physical activity^39,40^) pathways. We then tested whether associations could be replicated in a second longitudinal sample (hereafter, *study 2*), where we investigated associations over 14 years between adolescent volunteering and diabetes diagnosis in adulthood among Black adolescents in a national dataset. Across both studies, we hypothesized that adolescents who volunteered, and volunteered more frequently, would have better CMH (lower rates of metabolic syndrome, diabetes) compared to those who did not volunteer.

## Methods

This cohort study used data from 2 separate studies. Study 1 was approved by Northwestern University’s institutional review board. All participants provided written assent or consent, depending on age. All caregivers provided written consent. Study 2 was approved by the University of North Carolin’s institutional review board. Participants provided written informed consent. Data from this study are publicly available. Approval to conduct analyses on these data was obtained from the University of Georgia’s institutional review board. This study is reported following the Strengthening the Reporting of Observational Studies in Epidemiology (STROBE) reporting guideline.

### Study 1

#### Design, Setting, and Participants

Study 1 was a cross-sectional study. Data collection spanned November 19, 2018, to December 16, 2022. Participants were youths who self-reported as Black race, ages 14 to 19 years, recruited from the greater Chicago, Illinois area. Families were from lower-income households (ie, <2 times the federal poverty threshold). Eligible youths completed psychosocial questionnaires and health measures including a fasting blood draw (eMethods 1 in Supplement 1).

#### Measures

##### Volunteering

Volunteering was defined as “unpaid work, e.g., community service, intended to help others,” consistent with previous research recommendations.^41^ The first question asked whether youths had volunteered in the past 6 months (yes or no). The second question asked how often they volunteered.

##### Metabolic Syndrome

Metabolic syndrome was diagnosed according to International Diabetes Federation guidelines,^42^ based on clinical cutoff levels established for components of waist circumference, blood pressure, cholesterol, and glucose. Because of the young age of this sample, only 4% met criteria for metabolic syndrome diagnosis. Hence we focused analyses on 2 metabolic syndrome outcomes, consistent with previous research on younger samples.^43,44,45^ One was a count of the number of metabolic syndrome components for which participants were above clinical cutoff levels, consistent with previous research.^44,46,47,48,49^ Second, acknowledging concerns about the validity of dichotomizing youths into risk categories when metabolic functioning is distributed on a continuum,^50,51^ a metabolic syndrome composite was calculated using the mean of *z* scores for each metabolic syndrome component, consistent with previous research^43,44,45,52,53^.

#### Potential Mediators and Covariates

Possible pathways between volunteering and metabolic syndrome were probed, including physical activity (measured objectively via actigraphy), depressive symptoms,^54^ and purpose in life.^55^ Age, sex at birth, pubertal status, and family SES were included as covariates.

### Study 2

#### Design, Setting, and Participants

Study 2 was a longitudinal cohort study, spanning 1994 to 2008. Data were drawn from the National Longitudinal Study of Adolescent Health (Add Health), a nationally representative sample of US adolescents in grades 7 to 12 in 1994 to 1995. For replicability purposes, the sample for this analysis was restricted to participants who self-reported as non-Hispanic Black, with family income $30 000 or less (ie, ≤2 times the federal poverty threshold for family of 4 in 1994), and complete data on study variables (eMethods 2 in Supplement 1).

#### Measures

##### Volunteering

At wave 3 (mean age, 22), participants were asked: “At any time during your adolescence, when you were between 12-18 years old, did you regularly participate in volunteer or community service work?” Response options were yes or no.

##### Diabetes

At wave 4 (mean age, 29), researchers collected whole blood spots for analysis of glycosylated hemoglobin and glucose. Criteria for diabetes are in eMethods 2 in Supplement 1 and consistent with previous Add Health studies.^56^

#### Covariates

Covariates included age, sex, body mass index, parent report of adolescent diabetes diagnosis, and family SES. All covariate variables were collected at wave 1.

### Statistical Analysis

*P* values were 2-sided, and statistical significance was set at *P* ≤ .05. Data were analyzed from April 2024 to November 2025.

#### Study 1

For analyses with metabolic syndrome composite score, univariate analyses of covariance were used to test differences between those who did vs did not volunteer. For associations between volunteering frequency and metabolic syndrome composite, hierarchical multiple linear regression analyses were conducted, with covariates entered in step 1, and volunteering frequency entered in step 2. Parallel analyses were conducted for associations with potential mediators.

Metabolic syndrome count score was a count dependent variable (mean [SD], 0.94 [0.90]; variance, 0.81). Hence, Poisson analyses of covariance were used to test differences between participants who did vs did not volunteer. To test associations of volunteering frequency and potential mediators with metabolic syndrome count, Poisson regression analyses were conducted.

For mediation analyses with metabolic syndrome composite, we used the Hayes PROCESS macro, a regression-based approach.^57^ For mediation analyses with metabolic syndrome count, we used path analyses in Mplus version 8.2 (given Poisson regressions).

#### Study 2

Analyses were performed in Mplus version 8.2 with sampling weights from wave 4. Associations were tested using mixed-effect logistic regressions.

## Results

### Study 1

#### Descriptive Information and Preliminary Analyses

Study 1 included 400 Black adolescents (256 [64.0%] female; mean [SD] age, 16.39 [1.56] years), with 196 participants (49.0%) reporting volunteering in the past 6 months (Table 1). Univariate analyses (no covariates included) found that participants who volunteered had higher metabolic syndrome composites than those who did not (*t* = 2.23; *P* = .03) (Table 1). Volunteering more frequently was correlated with higher metabolic syndrome composites (*r* = −0.10; *P* = .04). No associations were found with metabolic syndrome counts (volunteering as binary variable: *b* = −0.09; SE, 0.10; *P* = .37; volunteering frequency: *b* = −0.04; se, 0.06; *P* = .54).

**Table 1.** Descriptive Information for Study Samples

Characteristic	Participants, No. (%)	
	Volunteers	Nonvolunteers
**Study 1**			
No.	196	202
Age, mean (SD), y	16.45 (1.53)	16.34 (1.60)
Sex		
Female	130 (66.3)	126 (62.4)
Male	66 (33.7)	76 (37.6)
Pubertal status		
Early or midpuberty	30 (15.3)	31 (15.4)
Late puberty	94 (48.0)	104 (51.7)
Postpuberty	72 (36.7)	66 (32.8)
Log (Family SES), mean (SD)	0.47 (0.08)	0.46 (0.07)
Frequency of volunteering, mean (SD)	1.58 (0.70)	NA^a^
Log (MVPA), mean (SD), min/d	1.23 (0.42)	1.13 (0.54)
Depressive symptoms, mean (SD)	11.76 (5.58)	11.35 (6.15)
Purpose in life, mean (SD)	3.26 (0.48)	3.10 (0.57)^a^
Metabolic syndrome count, No.^b^		
0	81 (41.8)	73 (36.3)
1	58 (29.9)	70 (34.8)
2	49 (25.3)	46 (22.9)
3	6 (3.1)	12 (6.0)
Metabolic syndrome composite, mean (SD)^c^	−0.08 (0.56)	0.04 (0.52)^a^
**Study 2**			
No.	392	587
Age (wave 1), mean (SD), y	15.83 (1.76)	15.89 (1.67)
Sex		
Female	233 (59.4)	337 (57.4)
Male	159 (40.6)	250 (42.6)
Family SES (wave 1), mean (SD)	−0.74 (1.54)	−1.06 (1.41)
Log BMI (wave 1), mean (SD)	1.37 (0.09)	1.36 (0.08)
Diabetes status (wave 1)	1 (0.3)	4 (0.7)
Depressive symptoms (wave 4), mean (SD)	3.21 (2.59)	3.13 (2.88)
Physical activity (wave 4), mean (SD)	4.12 (5.26)	3.21 (4.70)^d^
Diabetes diagnosis (wave 4)	42 (10.7)	93 (15.8)^d^

Abbreviations: BMI, body mass index (calculated as weight in kilograms divided by height in meters squared); MVPA, minutes per day of moderate to vigorous physical activity; NA, not applicable; SES, socioeconomic status (standardized composite of family income and savings, log transformed in study 1; standardized composite of parent education, parent occupation, and household income in study 2).

^a^ *P* < .05.

^b^ Defined as the number of metabolic syndrome components above clinical cutoff criteria.

^c^ Defined as the mean *z* scores of each metabolic syndrome component.

^d^ *P* < .01 for difference between groups.

#### Volunteering and Metabolic Syndrome

**Figure 1.**
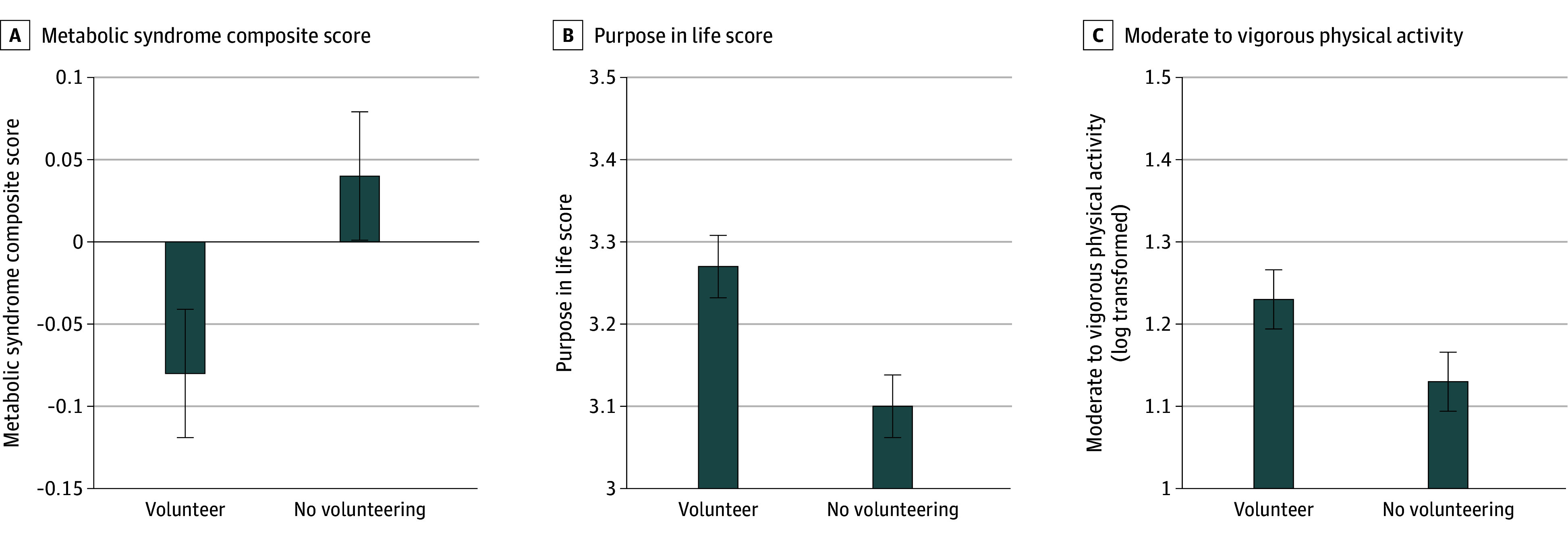
Differences Between Youths Who Volunteered vs Those Who Did Not Metabolic syndrome composite score is the mean of *z* scores of each metabolic syndrome component. Data are from study 1. Analyses control for age, sex, pubertal status, and family socioeconomic status. Estimated marginal means with standard error bars are depicted.

After adjusting for covariates, adolescents who volunteered had a 0.11-SD lower score on the metabolic syndrome composite (estimated marginal mean [EMM], −0.08; SE, 0.04) compared with those who did not volunteer (EMM, 0.04; SE, 0.04) (*F* = 4.51; *P* = .03) (Figure 1). Regression analyses demonstrated that for every 1-SD increase in volunteering frequency, there was a 0.11-SD decrease in metabolic syndrome composite scores (*b* = −0.06; SE, 0.03; *P* = .03).

After adjusting for covariates, adolescents who volunteered (EMM, 0.87; SE, 0.07) vs those who did not (EMM, 0.97; SE, 0.07) did not differ on metabolic syndrome counts (Wald × 2 = 1.13; *P* = .29). There was no association between volunteering frequency and metabolic syndrome counts (*b* = −0.04; SE, 0.06; *P* = .45).

#### Volunteering and Potential Mediator Variables

Adolescents who volunteered reported greater purpose in life (estimated marginal mean, 3.27; SE, 0.04) than those who did not (EMM, 3.10; SE, 0.04) (*F* = 10.17; *P* = .002) (Figure 1). A higher frequency of volunteering was associated with greater purpose in life (*b* = 0.08; SE, 0.03; *P* = .005).

Adolescents who volunteered had greater physical activity (log-transformed EMM, 1.23; SE, 0.04) than those who did not (EMM, 1.13; SE, 0.04) (*F* = 3.89; *P* = .049) (Figure 1). A higher frequency of volunteering was associated with greater physical activity (*b* = 0.06; SE, 0.03; *P* = .04). Volunteering was not associated with depressive symptoms (*F* = 0.27; *P* = .60; *b* = 0.38; SE, 0.32; *P* = .23).

#### Potential Mediator Variables and Metabolic Syndrome

Higher purpose in life was associated with lower metabolic syndrome composite (*b* = −0.16; SE, 0.05; *P* = .002) and with lower metabolic syndrome counts (*b* = −0.24; SE, 0.10; *P* = .01). There was no association between physical activity and metabolic syndrome composite (*b* = −0.11; SE, 0.06; *P* = .08). No association was found between physical activity and metabolic syndrome counts (*b* = −0.18; SE, 0.12; *P* = .15). Depressive symptoms were not associated with metabolic syndrome (composite: *b* = 0.00; SE, 0.00; *P* = .69; counts: *b* = 0.00; SE, 0.01; *P* = .86).

#### Mediation Analyses

**Figure 2.**
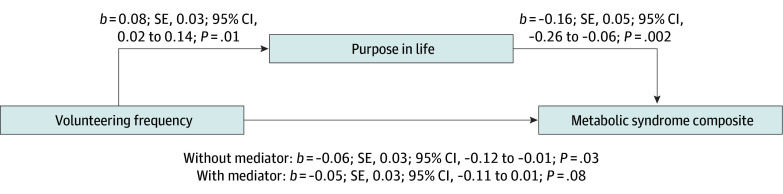
Psychological Pathway From Volunteering to Metabolic Syndrome Metabolic syndrome composite score is the mean of *z* scores of each metabolic syndrome component, controlling for age, sex, pubertal status, and family socioeconomic status in study 1. Without purpose in life in the model, the association between volunteering and metabolic syndrome was significant; however, once purpose in life is included, there was no longer a significant direct association from volunteering to metabolic syndrome.

The indirect pathway of volunteering (yes or no) to purpose in life to metabolic syndrome composite was significant (*b* = −0.03; 95% CI, −0.06 to −0.004; proportion of mediation, 22.4%), as was the indirect path of volunteering frequency to purpose in life to metabolic syndrome composite (*b* = −0.01; 95% CI, −0.03 to −0.002; proportion of mediation, 19.0%) (Figure 2). The indirect pathway of volunteering (yes or no) to purpose in life to metabolic syndrome counts was significant (*b* = −0.04; 95% CI, −0.10 to −0.01; proportion of mediation, 36.7%), as was volunteering frequency to purpose in life to metabolic syndrome counts (*b* = −0.02; 95% CI, −0.05 to −0.004; proportion of mediation, 45.2%).

In contrast, there were no significant indirect associations through depressive symptoms or physical activity. There was also no support for alternative mediation models (purpose in life to volunteering to metabolic syndrome) (eResults 1 in Supplement 1).

### Study 2

#### Descriptive Information

Study 2 included 979 Black adolescents (570 [58.2%] female; mean [SD] age, 15.87 [1.71] years), with 392 (40.0%) reported having volunteered regularly during adolescence. A total of 135 participants (13.8%) met criteria for diabetes at wave 4 (Table 1). eTable 1 in Supplement 1 presents differences between the study 2 sample and the larger Add Health sample.

#### Volunteering and Diabetes

After controlling for covariates, youths who volunteered during adolescence had lower odds of adult diabetes diagnosis (EMM, 9%, SE, 0.03) compared with those who did not volunteer (EMM, 18%; SE, 0.02; odds ratio, 0.48; 95% CI, 0.24-0.94; *P* = .03) (Table 2). Including additional sociodemographic, health status, and health behavior covariates did not change the significance of this finding (eTable 2 in Supplement 1). Including wave 4 covariates did not change this finding (eTable 3 in Supplement 1).

**Table 2.** Associations Between Volunteering in Adolescence and Diabetes at Ages 24-32 Years in Study 2 Cohort

Variable	OR (95% CI)	*P* value
Sex, male	1.01 (0.68-1.50)	.98
Age	1.08 (0.89-1.30)	.44
Family SES (ages 11-20 y)	0.95 (0.71-1.25)	.70
Log BMI (ages 11-20 y)	4.21 (0.95-18.68)	.06
Diabetes status (ages 11-20 y)	0.94 (0.07-11.97)	.96
Volunteering (ages 12-18 y)	0.48 (0.24-0.94)	.03

Abbreviations: BMI, body mass index (calculated as weight in kilograms divided by height in meters squared); OR, odds ratio; SES, socioeconomic status.

Purpose in life was not measured in Add Health, so we were not able to test the replicability of mediation findings in study 1. However, consistent with study 1, no evidence for mediation was found for depressive symptoms or physical activity (eResults 2 in Supplement 1).

## Discussion

To our knowledge, this cohort study is the first study to report—across 2 independent samples—that volunteering during adolescence was associated with better CMH (ie, lower adolescent metabolic syndrome composite, lower odds of adult diabetes). Patterns emerged in a group that experiences marginalization (Black adolescents from lower-income households). Dosage associations were found, such that those who volunteered more frequently had lower metabolic syndrome composite scores. Statistical mediation analyses revealed that higher purpose in life served as 1 pathway between volunteering and metabolic syndrome. In study 2, longitudinal analyses found that results held after controlling for wave 1 body mass index and adolescent diabetes diagnosis. Overall, our results suggest that positive aspects of SDOH are important to incorporate into health disparities models. One such component that merits further investigation is volunteering. Activities intended to benefit others may also be associated with better CMH in the helper.

Our findings are consistent with previous literature in adults showing associations between volunteering and metabolic syndrome, CVD, and mortality.^18,21,22^ Findings are also consistent with previous literature showing dosage patterns of more hours of volunteering being associated with better health in adults.^18,23,58^ We are aware of only 1 previous study documenting links between volunteering and biological outcomes during adolescence^29^; however, that study was not focused on clinical outcomes, unlike our study. Given that diseases, like CVD, involve physiological processes (eg, atherosclerosis) that begin during childhood and that track into adulthood,^26,59,60^ it is important to identify factors associated with CMH during earlier periods of life. Two previous studies of young adults did not find associations of volunteering with health outcomes^27,28^; however those studies did not focus on marginalized populations. It is possible that the associations of volunteering with health in younger populations first appear in marginalized groups (that are already at risk for poorer health) and then emerge in the general population later in adulthood.

Most previous studies on volunteering included largely White participants and those higher in SES. Groups that have experienced economic and social marginalization may have less available time and resources to volunteer. Nonetheless, individuals with lower SES tend to place more importance on relationships with others and are more oriented toward helping others,^61,62,63^ perhaps because connections between people are an important commodity when fewer material resources are present. While some might hypothesize that volunteering represents a burden for individuals with low SES, we found no evidence of this. On the contrary, we found beneficial associations between volunteering and health across 2 low-income samples. Given the striking disparities that exist by race and SES in CVD,^64,65,66^ there is a need to better understand factors that could mitigate the burden of such diseases in groups experiencing disadvantage. While there are numerous structural causes of health inequalities, this study’s findings nonetheless suggest that volunteering may be one beneficial activity that could be encouraged at the social network level, as our society simultaneously works toward structural solutions for health inequalities.

Why might volunteering be beneficial for CMH? We tested several pathways in study 1 and found that volunteering was associated with metabolic syndrome through a greater sense of purpose and meaning in life. Adolescence is a period when youths often develop a sense of purpose,^67^ and volunteering may provide a structured way of finding meaning during this period. Previous research has found associations of volunteering with purpose in life in adults^17,19,68^ and has found evidence for purpose in life as a pathway between volunteering and mental and physical health in adults.^37,38^ Furthermore, associations of volunteering with adolescent development are only apparent when adolescents reflect on their volunteering experiences.^69^ With respect to physical health, greater purpose in life has been associated with lower inflammation, CVD, and mortality in adults.^70,71,72^

In contrast, there was no support in study 1 or 2 for physical activity or depressive symptoms as mediators. Previous studies have found support for physical activity pathways between volunteering and mental or physical health in older adults.^17,18,21,39,40,73,74^ It is possible that in older populations, volunteering allows opportunities for physical activity at a time in life when it is common to become more sedentary. In contrast, adolescents have structured opportunities for physical activities (eg, sports teams, gym class), and thus may not need volunteering to stay active.

Similarly while previous studies found support for depressive symptoms pathways linked to volunteering and mental or physical health,^13,14,75^ these were also studies of older adults. It may be that in adolescents, other factors drive depressive symptoms more strongly, such as peer^76,77^ and family relationships.^78,79^

In terms of clinical significance, our studies found that volunteering during adolescence was associated with a more than 50% reduction in the odds of adult diabetes, and furthermore, for each 1-SD increase in volunteering frequency, there was a 0.11-SD decrease in metabolic syndrome score. Additionally, purpose in life accounted for 19% to 45% of the variance in the volunteering-metabolic syndrome relationship, suggesting that these pathways to CMH may be important to address in SDOH and health disparities research.

### Strengths and Limitations

Strengths of this study include the focus on a group experiencing marginalization, the emphasis on understanding CMH during earlier periods of life when prevention efforts can be implemented, and the use of 2 independent samples to replicate findings. Limitations include the cross-sectional nature of study 1, precluding conclusions about causality or directionality. Limitations of study 2 include not having measures of purpose in life to replicate study 1 findings. Additionally, study 2 is limited by a lack of repeated measures at every time point for volunteering, diabetes (objectively diagnosed), and covariates and by long intervals between assessments (when other factors linked to study outcomes might emerge). Other limitations more generally include volunteering being assessed retrospectively, the lack of details about volunteering (eg, type, length), and that other unmeasured variables may confound associations. Additionally, selection biases (eg, interest in research, time availability) may shape who agreed to participate. Future studies should test broader models situating volunteering within other social network (eg, work environments) and structural- or societal-level factors (eg, social service policies) that comprise SDOH and that contribute to health disparities. Furthermore, because casual conclusions cannot be reached without experimental designs, future research should conduct intervention trials of volunteering’s effects on CMH.

## Conclusions

In this cohort study of 2 independent samples of Black adolescents from lower-income households, a positive component of SDOH—volunteering—was associated with lower adolescent metabolic syndrome and lower odds of adult diabetes. Mediation analyses found that the association with volunteering operated via helping adolescents experience greater purpose in life. These findings suggest that encouraging adolescents to volunteer may represent one novel approach to improving CMH earlier in life. Volunteering may reflect one important, but largely unexplored, component of SDOH.^80^ If so, the notion that volunteering could be beneficial not only for recipients of volunteering efforts, but also for volunteers themselves, suggests the possibility of creating a 2-way street of benefits through a single activity that could promote health across groups while also strengthening the social fabric across communities.

## Notes

Supplement 1.
eMethods 1.

eResults 1.

eMethods 2.

eResults 2.
**eTable 1.** Study 2 Participants vs. Add Health Participants Not Included in Study 2 Analyses **eTable 2.** Associations between Volunteering in Adolescence and Diabetes in Adulthood (ages 24-32 years) in Study 2 Cohort [Including Additional Covariates] **eTable 3.** Associations between Volunteering in Adolescence and Diabetes in Adulthood (ages 24-32 years) in Study 2 Cohort [Including Wave 4 Covariate] **eFigure.** Broader Social Framework and Theoretical Model
eReferences.

Supplement 2.
Data Sharing Statement
