# Supplementary material for: Volunteering and Metabolic Syndrome and Diabetes in Black Adolescents From Low-Income Families
Source: JAMA Netw Open. 2026 Jan 12;9(1):e2553419. doi: 10.1001/jamanetworkopen.2025.53419 (PMC12797099; doi:10.1001/jamanetworkopen.2025.53419)
Supplement: Supplement 1. — eMethods 1. eResults 1. eMethods 2. eResults 2. eTable 1. Study 2 Participants vs. Add Health Participants Not Included in Study 2 Analyses eTable 2. Associations between Volunteering in Adolescence and Diabetes in Adulthood (ages 24-32 years) in Study 2 Cohort [Including Additional Covariates] eTable 3. Associations between Volunteering in Adolescence and Diabetes in Adulthood (ages 24-32 years) in Study 2 Cohort [Including Wave 4 Covariate] eFigure. Broader Social Framework and Theoretical Model eReferences. [file jamanetwopen-e2553419-s001.pdf]

Supplemental Online Content

Chen E, Germer SO, Moon H, Dezil J, Hayen R, Yu T. Volunteering and metabolic syndrome and diabetes in Black adolescents from low-income families. *JAMA Netw Open*. 2026;9(1):e2553419. doi:10.1001/jamanetworkopen.2025.53419

eMethods 1.

eResults 1.

eMethods 2.

eResults 2.

eTable 1. Study 2 Participants vs. Add Health Participants Not Included in Study 2 Analyses

eTable 2. Associations between Volunteering in Adolescence and Diabetes in Adulthood (ages 24-32 years) in Study 2 Cohort [Including Additional Covariates]

eTable 3. Associations between Volunteering in Adolescence and Diabetes in Adulthood (ages 24-32 years) in Study 2 Cohort [Including Wave 4 Covariate]

eFigure. Broader Social Framework and Theoretical Model

eReferences.

This supplemental material has been provided by the authors to give readers additional information about their work.

© 2026 Chen E et al. *JAMA Network Open*.

STUDY 1 eMETHOD

Study Design

This is a cross-sectional study, with data collection spanning 11/19/18-12/16/22. STROBE reporting guidelines were followed.

Participants

Participants were 400 Black youth ages 14 to 19 recruited from the greater Chicago area through advertisements, presentations at schools, outreach to community organizations, and through a direct mail campaign. The sample size was determined based on a power analysis conducted for the primary aims of the broader study, which were related to understanding predictors of combined academic and health trajectories in Black youth from lower-income households. Study inclusion criteria included youth who self-identified as Black, were between ages 14 and 19, and whose family reported their income to be below two times the federal poverty threshold for their household size. Other eligibility criteria included being English speaking, having no current major chronic illnesses that necessitated taking regular medication, having no mental health disorder serious enough to warrant hospitalization in the past year, and having no pervasive developmental disorder that would make the youth unable to complete the study protocol. Participants who were currently pregnant or acutely ill were offered the option of rescheduling.

Eligible youth were invited for a laboratory visit, during which youth completed psychosocial questionnaires and health measures including a fasting blood draw (scheduled for the morning, generally between 8-10am, to minimize diurnal variation). Youth provided either

© 2026 Chen E et al. *JAMA Network Open*.

written assent or consent (depending on age), and caregivers provided written consent for all study procedures, which were approved by the Northwestern University Institutional Review Board.

Measures

Volunteering. Volunteering was defined as “unpaid work, e.g., community service, intended to help others,” consistent with previous research recommendations[1]. The first question asked whether youth had volunteered in the past 6 months (yes/no). The second question asked how often they volunteered. With behaviors that can have a wide range of frequencies (e.g., where people can engage in a behavior anywhere from daily to weekly to monthly to yearly), it can be difficult to specify a unit when asking for a number amount (because for some, answering with number of times per week would make sense whereas for others answering with number of times per year would make the most sense). In addition, adolescents have been found to be more likely to overreport frequencies compared to adults when asked to report the number of times they engaged in a health behavior [2]. As a result, for many health-relevant behaviors, including volunteering, response categories are used [3]. The present study utilized the following response categories: 0=does not volunteer; 1=volunteers less than monthly; 2=volunteers at least monthly but less than weekly; 3=volunteers weekly. These response categories parallel and are commonly found in health behavior research for behaviors that can have wide ranges of frequencies, including marijuana use, opioid use/withdrawal episodes, and fast food consumption [4-7]. Two participants did not answer the volunteering questions.

© 2026 Chen E et al. *JAMA Network Open*.

MetS. Resting blood pressure was monitored with an automated auscultatory device (Carescape V100; GE) while the participant sat quietly. After a four minute acclimation, four readings were taken every 2 minutes, and the average of the last three readings was calculated. Waist circumference was measured at the midpoint of the upper iliac crest and lower costal margin, at the midaxillary line. Fasting blood was drawn into serum separator tubes. Serum was assayed at NorthShore University Health System's Core Laboratory for glucose and a cholesterol panel (visit times always scheduled in the morning to minimize diurnal variation).

MetS was diagnosed according to International Diabetes Federation guidelines[8]. These criteria specify that the diagnosis of MetS requires central adiposity, which for the participants in this sample is defined as waist circumference ≥94 cm for males and ≥80 cm for females. At least two of four additional components must also be present. This includes: (a) signs of early hypertension (systolic pressure ≥130 or diastolic pressure ≥85 mm Hg), (b) elevated triglycerides (≥150 mg/dL), (c) elevated fasting glucose (≥100 mg/dL), or (d) lowered high-density lipoprotein levels (<40 mg/dL in males and <50 mg/dL in females).

Because of the young age of this sample, only 4% met criteria for MetS diagnosis. Hence we focused analyses on two MetS outcomes, consistent with previous research on younger samples[9-11]. One was a count of the number of MetS components (the criteria listed above) for which participants met clinical cutoff criteria. This was used as an outcome because even though very few in the sample met formal diagnostic criteria for MetS, 61% (n=241) were already showing values above clinical cutoffs on at least 1 measure that is part of the MetS diagnostic criteria. The rationale is that the more criteria one is above clinical cutoffs for, the unhealthier that person's profile is and the closer they are to a diagnosis of MetS. This approach

© 2026 Chen E et al. *JAMA Network Open*.

has been used across multiple studies in previous research [10, 12-15]. Second, acknowledging concerns about the validity of dichotomizing youth into risk categories when metabolic functioning is distributed on a continuum[16, 17], a MetS composite was calculated using the mean of z-scores of each MetS component. The z-score composite is an established approach that has been used across multiple samples of adolescents in previous research[9-11, 18, 19], and that has been found to prospectively predict cardiovascular events into adulthood[18]. Four participants did not have sufficient data to calculate MetS scores.

Potential mediators. Possible pathways between volunteering and Mets were probed, including physical activity, depressive symptoms, and purpose in life (see eFigure 1).

Physical activity was measured using the Actigraph wGT3X-BI (Actigraph, Florida). Youth were asked to wear an actigraphy watch on their non-dominant wrist for 8 consecutive days, removing it only for showering or swimming. Motion was monitored in 1-min epochs using a triaxial accelerometer. Accelerometers are considered to provide more objective and direct measures of physical activity, as they minimize issues of recall or response bias that are found in self-report measures [20]. One week is considered an adequate time frame for determining habitual physical activity [21, 22]. Activity was computed by measuring duration of sedentary, light, moderate, and vigorous activity each day. Activity categories were determined using Chandler cutpoints [23], which have been used in other adolescent studies [24, 25]. Consistent with the fact that the Centers for Disease Control provides physical activity recommendations in terms of minutes of moderate or vigorous physical activity, the number of minutes in spent in either moderate or vigorous activity was totaled for each day, and then averaged across the week. 42 participants did not wear the watch for enough days to calculate physical activity levels.

© 2026 Chen E et al. *JAMA Network Open*.

Depressive symptoms were measured with the Center for Epidemiological Studies – Depression (CES-D) scale[26]. The CES-D scale is a widely used, validated scale administered in many survey studies that includes 10 items assessing depressive symptoms over the past week rated on a 0 to 3 scale (Cronbach's alpha=0.79). Responses were summed, and higher scores indicate the experience of greater depressive symptoms. Two participants were missing data on this measure.

Purpose in life was measured using the Persist subscale of the Shift-and-Persist Questionnaire [27]. This is a validated questionnaire that assesses participants' ability to cope with stressful life circumstances using 7 items rated on a 1 to 4 scale. The Persist subscale measures the ability to endure adversities by finding meaning and maintaining purpose in life (Cronbach's alpha=0.70). Responses across items were averaged, and higher scores indicate experiencing greater purpose in life. One participant was missing data on this measure.

Covariates. Youth age, sex at birth (0=female, 1=male), pubertal status, and family socioeconomic status (SES) were included as covariates. Pubertal status was measured using a questionnaire, the Pubertal Development Scale[28], which has been validated against the Tanner physical examination evaluation for determining pubertal stage. Pubertal stage is scored from 1=pre-pubertal to 5=post-pubertal (3 participants were missing data). Family SES was calculated by standardizing and averaging caregiver reports of family income and family savings (2 participants missing data).

Statistical Analyses

Distributions of study variables were first checked for normality. For the MetS composite score, there was one extreme outlier (>7SD), which was removed. Without this outlier, the MetS

composite score was normally distributed (skewness=0.66, kurtosis=0.51). The SES variable also had one extreme outlier (15 SD), which was removed. Both the SES and the physical activity variables were not normally distributed, so were log transformed. For pubertal status, there were 0 participants in the pre-pubertal stage, and only 6 participants who were in the early puberty stage; hence early and mid-puberty were combined into one category. Thus for the purposes of these analyses, pubertal status was comprised of 3 categories (early/mid puberty, late puberty, post puberty, dummy coded with post puberty as the reference category).

Volunteering frequency was an ordinal independent variable. For analyses, the likelihood ratio, Bayesian Information Criterion (BIC), and Akaike Information Criterion (AIC) tests described by Williams and Quiroz[29] were used to test whether the assumption of linear effects was justified. Two models were estimated. In the constrained model, the ordinal volunteering frequency predictor was treated as continuous with linear effects, and in the unconstrained model, the ordinal predictor was treated as categorical, so that effects need not be linear. The likelihood-ratio test X^2 values for the differences between the models were not statistically significant: $X^2(2) = 1.09$, $p = 0.58$ when using MetS composite as the dependent variable with a linear regression model; $X^2(2) = 1.27$, $p = 0.53$ when using MetS count as the dependent variable with a Poisson regression model. BIC and AIC values were also both lower when volunteering frequency was treated as continuous (BIC = 646.86 and AIC = 638.91 for MetS composite as the dependent variable; BIC = 996.41 and AIC = 988.45 for MetS count as the dependent variable) than when it was treated as categorical (BIC = 657.73 and AIC = 641.82 for MetS composite; BIC = 1007.10 and AIC = 991.18 for MetS count). These results suggested that

© 2026 Chen E et al. *JAMA Network Open*.

the more parsimonious model that treated volunteering frequency as a continuous predictor was preferable.

The MetS composite score was a continuous dependent variable. For analyses, univariate analyses of covariance (ANCOVAs) were used to test for differences between those who did versus did not volunteer on the MetS composite score (including covariates of age, sex, SES, and pubertal status). To test associations between volunteering frequency and MetS composite score, hierarchical multiple linear regression analyses were conducted, with covariates of age, sex, SES, and pubertal status entered in step 1, and then the volunteering frequency score entered in step 2. For associations of volunteering with potential mediators (physical activity, depressive symptoms, and purpose in life), parallel ANCOVAs and multiple regression analyses were conducted as described above, except with each mediator (instead of MetS) as the outcome variable. For associations of potential mediators with MetS composite, hierarchical multiple linear regression analyses were conducted in which the MetS composite score was regressed upon covariates in step 1, and then the potential mediator in step 2.

MetS count score was a count dependent variable variable (mean = 0.94, SD = 0.90, variance = 0.81). The distribution of this variable does not indicate overdisperson, and hence a Poisson analytic approach is appropriate when MetS count score is the dependent variable. For analyses, Poisson ANCOVAs were used to test for differences between those who did versus did not volunteer on the MetS count score (including covariates of age, sex, SES, and pubertal status). To test associations of volunteering frequency and potential mediators with MetS count score, Poisson regression analyses were conducted.

© 2026 Chen E et al. *JAMA Network Open*.

For mediation analyses (volunteering→mediator→MetS) using MetS composite as the outcome, the Hayes PROCESS macro was used, a regression-based approach that calculates the indirect effect as a product of the regression coefficient between the independent variable and the mediator and the regression coefficient between the mediator and the dependent variable[30]. Nonparametric bootstrapping was used to obtain the bias-corrected and accelerated confidence intervals of parameter estimates for significance testing. The parameter estimate was calculated 5000 times using random sampling with replacement to build a sampling distribution[31]. For mediation analyses with MetS count as the outcome, the path analyses in Mplus 8.2 was used (given the use of Poisson regressions for this outcome variable). The association between the independent variable and the mediator was tested with linear regression, and the association between mediator and the dependent variable was tested with Poisson regression. The Model Indirect function was used to calculate the indirect effect with nonparametric bootstrapping. Covariates above were adjusted in all analyses. An alternate model of mediator→volunteering→MetS was also tested.

STUDY 1 eRESULTS

Additional Mediation Analyses

There were no significant indirect effects from volunteering to MetS through depressive symptoms (volunteering to MetS composite coefficient=0.000, 95%CI: -0.008, 0.006; frequency of volunteering to MetS composite=0.000, 95%CI: -0.006, 0.004; volunteering to MetS counts=0.000, 95%CI: -0.016, 0.008; volunteering frequency to MetS counts=0.000, 95%CI: -0.013, 0.006). There were also no significant indirect effect from volunteering to MetS through physical activity (volunteering to MetS composite=-0.009, 95%CI: -0.029, 0.003; volunteering

© 2026 Chen E et al. *JAMA Network Open*.

frequency to MetS composite=-0.005, 95%CI: -0.016, 0.002; volunteering to MetS counts=-0.016, 95%CI: -0.061, 0.003; volunteering frequency to MetS counts=-0.009, 95%CI: -0.032, 0.002).

Given support for only purpose in life as a mediator, we tested an alternate model whereby purpose in life was associated with MetS through volunteering. There was no significant indirect effect from purpose in life to volunteering (y/n) to MetS composite (coefficient=-0.062, 95%CI: -0.170, 0.008) or from purpose in life to volunteering frequency to MetS composite (coefficient=-0.013, 95%CI: -0.033, 0.002). There also was no significant indirect effect from purpose in life to volunteering (y/n) to MetS count (coefficient=-0.046, 95%CI: -0.223, 0.084) or from purpose in life to volunteering frequency to MetS count (coefficient=-0.006, 95%CI: -0.042, 0.022).

STUDY 2 eMETHOD

Study 2 was included as a replicability test of Study 1. We drew from an existing national publicly available dataset of U.S. adolescents who were followed into adulthood and had measures of both volunteering and diabetes.

Study Design

This is a longitudinal cohort study that spanned 1994-2008. STROBE reporting guidelines were followed.

Participants

Data were drawn from Waves 1, 3, and 4 of the National Longitudinal Study of Adolescent Health (Add Health), a nationally representative sample of adolescents in grades 7 through 12 in the United States in 1994-1995. At Wave 1, stage 1 involved recruitment of a

© 2026 Chen E et al. *JAMA Network Open*.

stratified random sample of all high schools in the US, along with a feeder middle school for each high school, for in-school surveys. In total, 144 schools were enrolled. Stage 2 of Wave 1 (1994-1995) involved recruiting an in-home data collection sample of 27,745 adolescents ages 11-20, based on student enrollment rosters from participating schools. At Wave 3 (2001-2002) 15,197 participants (ages 18 to 27 years) and at Wave 4 (2008) 14,800 participants (ages 24 to 32 years) took part in the follow-up in-home data collections. To make the sample as similar as possible to Study 1 for replication purposes, the analytic sample for this study was restricted to participants who self-reported as non-Hispanic Black, who had a family income (reported by parents) to be at or below two times the federal poverty threshold for a 4-person household in 1994 ($30,000), and who had complete data on all study variables at Waves 1, 3, and 4 (n = 979). The mean ages of youth were 15.87 years (SD = 1.70) at Wave 1, 21.73 years (SD = 1.76) at Wave 3, and 28.76 years (SD = 1.74) at Wave 4.

Measures

Volunteering. At Wave 3, as part of the in-home questionnaire, youth were asked: At any time during your adolescence, when you were between 12 to 18 years old, did you regularly participate in volunteer or community service work (yes/no)? Of 979 participants, 392 (40.0%) reported having volunteered during adolescence.

Diabetes. At Wave 4, field researchers collected whole blood spots from a finger capillary prick onto filter paper for laboratory analysis of glycosylated hemoglobin (HbA1c) and glucose. HbA1c was measured using a Roche COBAS INTEGRA 700 Analyzer (Roche Diagnostics, Indianapolis, IN), and is an integrated measure of blood glucose control over the preceding 2 to 3 months. Sensitivity of the assay was 3%. Intra- and inter-assay CVs ranged from 2.2-2.4%.

© 2026 Chen E et al. *JAMA Network Open*.

Glucose concentrations were determined using a Synergy HT Microtiter Plate Reader (BioTek, Winooski, VT). Participants were not required to be fasting; hence there are fasting glucose concentrations for some participants, and non-fasting values for others. Sensitivity of the assay was 22 mg/dl; intra-assay CV was 4.4%, and inter-assay CV was 4.8%. In addition, participants were interviewed and asked whether a health care provider ever told them that they have or had diabetes. They were also asked about their use of prescription medications for diabetes during the past 4 weeks. Participants were classified as having diabetes if they had: a fasting glucose level ≥ 126 mg/dl, a non-fasting glucose level ≥ 200 mg/dl, an HbA1c level ≥ 6.5%, self-reported a history of diabetes except during pregnancy, or used diabetes medication during the past 4 weeks. These criteria are consistent with previous Add Health studies[32]. Of 979 participants, 135 (13.8%) were classified as having diabetes.

Covariates. Covariates included the age of the participants at Wave 1 as a continuous variable. Sex was coded as male (1) or female (0). At the Wave 1 in-home survey, participants reported their height and weight. This information was used to compute adolescents' Wave 1 body mass index (BMI). At Wave 1, parents reported whether the adolescent currently had diabetes (1) or not (0). At Wave 1, family SES was measured by a combination of parent education, parent occupation, and total household income[33]. Parent education (of both parents if it was a two parent household) was reported by a parent and coded from 0 (no formal education) to 7 (professional training beyond 4 years in college). Codes were averaged for two parent households. Parents also reported their total household income before taxes. This measure was coded continuously in dollars (thousand). Youths were asked to identify their parents' occupation from a list of 14 possible groupings ranging from 1 (farm/fishery worker) to

© 2026 Chen E et al. *JAMA Network Open*.

14 (professional/doctor/lawyer/scientist). Codes were averaged for two parent households. These three components were standardized and then summed to form the family SES composite.

Statistical Analysis

Distributions of study variables were first checked for normality. The BMI variable was not normally distributed and was log transformed.

Analyses were performed in Mplus 8.2[2], using Type=COMPLEX command with sampling weights from wave 4 and identifying the included study sample as a subpopulation of the original survey-weighted cohort. The models specified clustering at the school level to account for non-independence of observations among participants attending the same schools. Associations between volunteering and diabetes were tested using mixed-effect logistic regression analyses, with age, sex, family SES, BMI, and diabetes status at Wave 1 entered as covariates.

While our primary analyses – for the purposes of replication – included a parallel set of covariates as Study 1, we recognize that Add Health contains many other variables that could be included as covariates in these analyses. To check the robustness of findings, in supplemental analyses, we included a wide range of additional covariates, following other Add Health papers on similar topics[34]. These included sociodemographic variables, health status variables, and health behavior variables. The only variables that we did not include from the Nakamura paper above were: race/ethnicity (because our sample was all Black), parent variables (e.g., mother age, mother happiness because our study was not focused on parent factors), psychosocial and academic factors (because our study was not focused on academic factors and because

© 2026 Chen E et al. *JAMA Network Open*.

psychosocial factors were conceptualized as mediators in our study), and suicidal ideation (because of its overlap with depressive symptoms, one of our proposed mediators).

While our primary analyses included covariates at baseline (Wave 1), in supplemental analyses, we also included covariates that were measured again at Wave 4 (BMI) to test the robustness of results. We did not include other covariates at Wave 4 because follow-ups were done an equal number of years apart for all participants (so no need to include age again as a covariate), because sex did not change, and because family SES was not collected at Wave 4 (there was no parent report at this wave).

This dataset did not contain measures of purpose in life, so we were unable to test the replicability of the mediation analyses in Study 1. However, we did test whether the lack of mediation for depressive symptoms and physical activity from Study 1 replicated in this Add Health sample. Depressive symptoms was assessed at Wave 4 using 5 items rated on a 4 point scale asked over the past week (e.g., You felt sad). Physical activity was assessed at Wave 4 with 6 items rated on an 8 point scale about past week engagement in different types of physical activity (e.g., How many times did you participate in strenuous team sports such as football, soccer, basketball, lacrosse, rugby, field hockey, or ice hockey?).

STUDY 2 eRESULTS

Preliminary Analyses

The Add Health subsample that was included in these study analyses had differences from the larger Add Health sample who were not included in these analyses. Because the subsample was specifically drawn to be low SES, the family SES of our study subsample was lower than the family SES of the larger Add Health sample. In addition, our Add Health

© 2026 Chen E et al. *JAMA Network Open*.

subsample also was younger, more likely to be female, had higher BMI, was less likely to volunteer, and was more likely to have diabetes. See eTable 1.

Supplemental Analyses

After including additional sociodemographic, health status, and health behavior covariates, volunteering during adolescence continued to be associated with diabetes in adulthood, OR=0.46, 95% CI: 0.23, 0.91, p=.03. See eTable 2.

After including the Wave 4 covariate of BMI, volunteering during adolescence continued to be associated with diabetes in adulthood, OR=0.42, 95% CI: 0.23, 0.80, p=.008. See eTable 3.

Mediation Analyses

Volunteering was not associated with depressive symptoms, b=0.33, se=0.27, p=0.23. Depressive symptoms were not associated with diabetes diagnosis, OR=0.95, 95% CI [0.86, 1.04], p=0.28. There was no significant indirect effect from volunteering to diabetes diagnosis through depressive symptoms, coefficient=-0.017, 95%CI [-0.063, 0.029].

Volunteering also was not associated with physical activity, b=0.85, se=0.53, p=0.11. Physical activity was not associated with diabetes diagnosis, OR=0.95, 95% CI [0.89, 1.02], p=0.16. There was no significant indirect effect from volunteering to diabetes diagnosis through physical activity, coefficient=-0.040, 95%CI [-0.119, 0.038].

The lack of significant indirect effects for depressive symptoms and physical activity in this Add Health subsample parallels the lack of significant indirect effects for these same study variables in Study 1.

© 2026 Chen E et al. *JAMA Network Open*.

eTable 1. Study 2 Participants vs. Add Health Participants Not Included in Study 2 Analyses

	No. (%)	M (SD)	No. (%)	M (SD)
Wave 1	Study Sample (n = 979)		Not Included (n = 19745)	
Age		15.87 (1.71)		16.18 (1.72)***
Sex	Study Sample (n = 979)		Not Included (n = 19760)	
Female	570 (58.2)		9908 (50.1)***	
Male	409 (41.8)		9852 (49.9)	
	Study Sample (n = 979)		Not Included (n = 18827)	
Family SES		-0.93 (1.47)		0.05 (1.99)***
	Study Sample (n = 979)		Not Included (n = 19192)	
Log (BMI)		1.36 (0.09)		1.34 (0.08)***
	Study Sample (n = 979)		Not Included (n = 16544)	
Diabetes status	5 (0.5)		73 (0.4)	
Wave 3	Study Sample (n = 979)		Not Included (n = 14148)	
Volunteering status	392 (40.0)		6221 (44.0)*	
Wave 4	Study Sample (n = 979)		Not Included (n = 13820)	
Diabetes diagnosis	135 (13.8)		894 (6.5)***	

Note: SES=socioeconomic status (standardized composite of parent education, parent occupation, and household income). BMI=body mass index, log transformed. *p<.05, ***p<.001 for difference between groups.

© 2026 Chen E et al. *JAMA Network Open*.

eTable 2. Associations between Volunteering in Adolescence and Diabetes in Adulthood (ages 24-32 years) in Study 2 Cohort [Including Additional Covariates]

	Diabetes Status (ages 24-32)	
Predictors	OR (95% CI)	*p*
1. Sex, male	1.19 (0.69, 2.05)	.54
2. Age	1.03 (0.88, 1.21)	.72
3. Family SES (ages 11-20)	0.96 (0.70, 1.32)	.80
4. Log BMI (ages 11-20)	107.17 (3.20, 3592.89)	.009
5. Diabetes status (ages 11-20)	0.75 (0.01, 71.30)	.90
6. Volunteering (ages 12-18)	0.46 (0.23, 0.91)	.03
Additional covariates		
1. Nativity status	25334.04 (0.13, 4.79×10^{9})	.10
2. Region, West	0.92 (0.27, 3.71)	.90
3. Region, Midwest	0.25 (0.07, 0.84)	.03
4. Region, South	0.44 (0.13, 1.45)	.18
5. Single-parent family	0.80 (0.43, 1.50)	.49
6. Number of siblings	0.96 (0.88, 1.05)	.39
7. Family Welfare receipt	1.16 (0.56, 2.38)	.69
8. Health insurance status	1.00 (0.49, 2.05)	.99
9. Smoker in household	0.59 (0.29, 1.20)	.15
10. Self-rated health	1.06 (0.83, 1.35)	.63
11. Somatic symptoms	1.55 (0.47, 5.09)	.47
12. Health condition diagnosis	0.76 (0.52, 1.12)	.17
13. Functional limitations	0.45 (0.11, 1.95)	.29
14. Sleep disturbance	0.72 (0.54, 0.96)	.03
15. Physical inactivity	1.67 (0.87, 3.18)	.12
16. Cigarette smoking	1.00 (0.94, 1.06)	.99
17. Binge drinking	0.81 (0.64, 1.03)	.08
18. Marijuana use	1.00 (0.98, 1.01)	.53

© 2026 Chen E et al. *JAMA Network Open*.

19. Illicit drug use	1.00 (0.99, 1.01)	.99
20. Sexually transmitted infections	0.54 (0.14, 2.12)	.37
21. Preventative health care use	0.83 (0.46, 1.50)	.54

Note: SES=socioeconomic status. BMI=body mass index. OR=odds ratio. CI=confidence interval. Follow-up analyses were conducted by adjusting for a wide range of additional covariates including sociodemographic, health status, and health behaviors factors. Sociodemographic factors included nativity status (born in the United States, yes/no), geographic region (West, Midwest, South, Northeast), family structure (single-parent family, yes/no), number of siblings, household welfare receipt (yes/no), insurance status (time without health insurance, yes/no), and cigarette smoker in household (yes/no). Health status factors included somatic symptoms (such as headache, upset stomach, cold sweats, sore throat or cough, skin problems, dizziness, chest pains, etc), physical health condition (such as migraine headaches, asthma or emphysema, allergies or hay fever, etc), functional limitations (difficulty using hands, arms, legs, or feet because of a permanent physical condition, yes/no), and self-rated health (1=excellent to 5 = poor). Health behaviors factors included sleep disturbance (how often having trouble falling asleep or staying asleep, 0=never to 4=every day), physical inactivity (total past week physical activities ≤ 2, including sports involvement, cycling/skating, and general exercise, 0 = not at all to 3= 5 or more times), cigarette smoking (past month number of days smoked past month), binge drinking (past year number of days binge drinking of 5 or more in a row), marijuana use (past year number of times used marijuana) , illicit drug use (past month times of use illegal drugs), history of sexually transmitted infections [STIs] (such as Chlamydia, syphilis, genital herpes, HIV or AIDS, etc), and preventative health care use (past year having a routine physical examination, yes/no). All missing data in these covariates were handled with full information maximum likelihood (FIML) estimation.

© 2026 Chen E et al. *JAMA Network Open*.

eTable 3. Associations between Volunteering in Adolescence and Diabetes in Adulthood (ages 24-32 years) in Study 2 Cohort [Including Wave 4 Covariate]

	Diabetes Status (ages 24-32)	
Predictors	OR (95% CI)	*p*
1. Sex, male	1.24 (0.72, 2.14)	.44
2. Age	1.05 (0.90, 1.22)	.55
3. Family SES (ages 11-20)	0.96 (0.69, 1.32)	.79
4. Log BMI (ages 11-20)	5.53 (0.06, 490.12)	.46
5. Diabetes status (ages 11-20)	0.83 (0.01, 48.49)	.93
6. Volunteering (ages 12-18)	0.42 (0.23, 0.80)	.008
Additional covariates		
1. Nativity status	10126.04 (0.08, $1.34x10^6$)	.13
2. Region, West	0.81 (0.23, 2.92)	.75
3. Region, Midwest	0.24 (0.08, 0.72)	.01
4. Region, South	0.39 (0.13, 1.18)	.09
5. Single-parent family	0.78 (0.42, 1.45)	.43
6. Number of siblings	0.96 (0.88, 1.06)	.41
7. Family Welfare receipt	1.15 (0.58, 2.29)	.69
8. Health insurance status	0.93 (0.43, 2.01)	.85
9. Smoker in household	0.57 (0.28, 1.18)	.13
10. Self-rated health	1.06 (0.83, 1.34)	.66
11. Somatic symptoms	1.60 (0.47, 5.47)	.45
12. Health condition diagnosis	0.73 (0.50, 1.07)	.11
13. Functional limitations	0.50 (0.12, 2.07)	.34
14. Sleep disturbance	0.71 (0.53, 0.96)	.03
15. Physical inactivity	1.62 (0.85, 3.07)	.14
16. Cigarette smoking	1.00 (0.95, 1.06)	.93
17. Binge drinking	0.83 (0.66, 1.05)	.13

© 2026 Chen E et al. *JAMA Network Open*.

18. Marijuana use	1.00 (0.99, 1.01)	.54
19. Illicit drug use	1.00 (0.99, 1.01)	.92
20. Sexually transmitted infections	0.51 (0.12, 2.11)	.54
21. Preventative health care use	0.843 (0.48, 1.47)	.35
22. BMI (ages 24-32)	1.04 (1.01, 1.08)	.01

Note: See eTable 2 for explanations of covariates.

© 2026 Chen E et al. *JAMA Network Open*.

eFigure 1. Broader Social Framework & Theoretical Model. Top panel: Volunteering is hypothesized to be linked to health outcomes such as metabolic syndrome through cognitive, emotional, and health behavior pathways. Volunteering is conceptualized as an interpersonal behavior that connects the individual to a larger social network. Mechanisms of volunteering are theorized to operate through individual-level pathways to influence physical health. Bottom panel: The broader background of social network and structural/societal level factors comprise the social determinants of health and contribute to health disparities when factors disproportionately affect marginalized groups (such as those who are the focus of the present study, Black adolescents from lower-income households). Social network factors include (but not limited to) constructs such as family relationship characteristics, social support/social isolation, and work characteristics. Structural/societal level factors include (but not limited to) constructs such as structural racism, neighborhood crime, food deserts, health care access/quality, and social service policies. See [35-37] for more detailed discussions of health disparities frameworks and social exposome models.

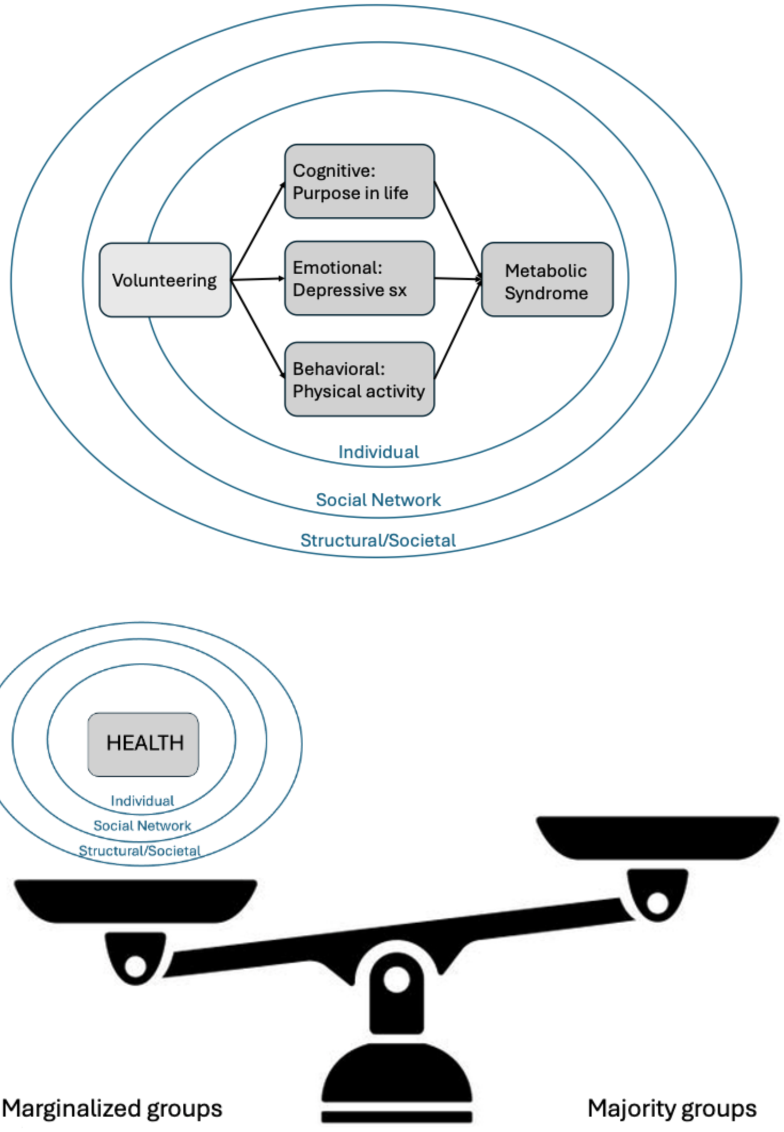

© 2026 Chen E et al. *JAMA Network Open*.

eReferences

1. Wilson J, Musick M. Who cares? Toward an integrated theory of volunteer work. *American Sociological Review*. 1997;62 694-713.
2. Mathiowetz NA, Dipko S. A comparison of response error by adolescents and adults. *Medical Care*. 2000;38 374-382.
3. Konrath S, Fuhrel-Forbis A, Lou A, Brown S. Motives for volunteering are associated with mortality risk in older adults. *Health Psychology*. 2012;31 87-96.
4. Ashrafioun L, Bishop TM, Conner KR, Pigeon WR. Frequency of prescription opioid misuse and suicidal ideation, planning, and attempts. *Journal of Psychiatric Research*. 2017;92 1-7.
5. Bluthenthal RN, Simpson K, Ceasar RC, Zhao J, Wenger L, Kral AH. Opioid withdrawal symptoms, frequency, and pain characteristics as correlates of health risk among people who inject drugs. *Drug and Alcohol Dependence*. 2020;211 107932.
6. Subbaraman MS, Barnett SB, Karriker-Jaffe KJ. Risks associated with mid level cannabis use among people treated for alcohol use disorder. *Alcoholism: Clinical and Experimental Research*. 2019;43 690-694.
7. Crawford GB, Khedkar A, Flaws JA, Sorkin JD, Gallicchio L. Depressive symptoms and self-reported fast-food intake in midlife women. *Preventive Medicine*. 2011;52 254-257.
8. Cornier MA, Dabelea D, Hernandez TL et al. The metabolic syndrome. *Endocrine Reviews*. 2008;29 777-822.

© 2026 Chen E et al. *JAMA Network Open*.

9. Levine CS, Markus HR, Austin MA, Chen E, Miller GE. Students of color show health advantages when they attend schools that emphasize the value of diversity. *Proceedings of the National Academy of Sciences*. 2019;116 6013-6018.

10. Miller GE, Chen E, Armstrong CC et al. Functional connectivity in central executive network protects youth against cardiometabolic risks linked with neighborhood violence. *Proceedings of the National Academy of Sciences*. 2018;115 12063-12068.

11. Lam PH, Chen E, Jiang T et al. Responsive parental support buffers the link between chronic stress and cardiometabolic risk among adolescents. *Brain Behavior and Immunity*. 2024;116 114-123.

12. Miller GE, Chen E, Yu T, Brody GH. Youth who achieve upward socioeconomic mobility display lower psychological distress but higher metabolic syndrome rates as adults: Prospective evidence from the National Study of Adolescent Health and the Midlife in the United States Study. *Journal of the American Heart Association*. 2020;9 e015698.

13. Ehrlich KB, Lyle SM, Corallo KL et al. Socioeconomic disadvantage and high-effort coping in childhood: Evidence of skin-deep resilience. *Journal of Child Psychology and Psychiatry*. 2024;65 358-364.

14. Chen E, Lam PH, Yu T, Brody GH. Racial disparities in school belonging and prospective associations with diabetes and metabolic syndrome. *JAMA Pediatrics*. 2023;177 141-148.

15. Miller GE, Lachman ME, Chen E, Gruenewald TL, Seeman TE. Pathways to resilience: Maternal nurturance as a buffer against childhood poverty's effects on metabolic syndrome at midlife. *Psychological Science*. 2011;22 1591-1599.

© 2026 Chen E et al. *JAMA Network Open*.

16. Goodman E. Metabolic syndrome and the mismeasure of risk. *Journal of Adolescent Health*. 2008;42 538-540.

17. Goodman E, Daniels SR, Morrison JA, Huang B, Dolan LM. Contrasting prevalence of and demographic disparities in the World Health Organization and National Cholesterol Education Program Adult Treatment Panel III definitions of metabolic syndrome among adolescents. *Journal of Pediatrics*. 2004;145 445-451.

18. Jacobs DR, Woo JG, Sinaiko AR et al. Childhood cardiovascular risk factors and adult cardiovascular events. *New England Journal of Medicine*. 2022;386 1877-1888.

19. Chen E, Kim J, Law J, Obi V, Gallivan SU, Hayen R. Superwoman schema and metabolic syndrome in Black adolescent girls. *Journal of Behavioral Medicine*. 2025;48 745-755.

20. Adamo KB, Prince SA, Tricco AC, Connor-Gorber S, Tremblay M. A comparison of indirect versus direct measures for assessing physical activity in the pediatric population: A systematic review. *International Journal of Pediatric Obesity*. 2009;4 2-27.

21. Trost SG, McIver KL, Pate RR. Conducting accelerometer-based activity assessments in field-based research. *Med Sci Sports Exerc*. 2005;37 (11 Suppl):S531-43.

22. Matthews CE, Hagströmer M, Pober DM, Bowles HR. Best practices for using physical activity monitors in population-based research. *Med Sci Sports Exerc*. 2012;44 (1 Suppl 1):S68-76.

23. Chandler JL, Brazendale K, Beets MW, Mealing BA. Classification of physical activity intensities using a wrist worn accelerometer in 8-12 year old children. *Pediatric Obesity*. 2015;11 120-127.

© 2026 Chen E et al. *JAMA Network Open*.

24. Cushing CC, Mitchell TB, Bejarano CM, Walters RW, Crick CJ, Noser AE. Bidirectional associations between psychological states and physical activity in adolescents. *Journal of Pediatric Psychology*. 2017;42 559-568.

25. Cespedes Feliciano EM, Quante M, Rifas-Shiman SL, Redline S, Oken E, Taveras EM. Objective sleep characteristics and cardiometabolic health in young adolescents. *Pediatrics*. 2018;142 e20174085.

26. Radloff LS. The CES-D scale: A self-report depression scale for research in the general population_. *Journal of Applied Psychological Measurement*. 1977;1 385-401.

27. Lam PH, Miller GE, Chiang JJ et al. One size does not fit all: Links between shift-and-persist on asthma in youth are moderated by perceived family social status and experience of unfair treatment. *Development and Psychopathology*. 2018;30 1699-1714.

28. Petersen AC, Crockett L, Richards M, Boxer A. A self-report measure of pubertal status: Reliability, validity, and initial norms. *Journal of Youth and Adolescence*. 1988;17_ 117-133.

29. Williams RA, Quiroz C. Ordinal regression models. In: Atkinson P, Delamont S, Cernat A, Sakshaug JW, Williams RA, eds. *SAGE Research Methods Foundations*. London: SAGE Publications; 2020:http://dx.doi.org/10.4135/9781526421036885901.

30. Preacher KJ, Hayes AF. SPSS and SAS procedures for estimating indirect effects in simple mediation models. *Behav Res Methods Instrum Comput*. 2004;36 (4):717-731.

31. Hayes AF. *Introduction to mediation, moderation, and conditional process analysis, Second Edition*. New York, NY: Guilford Press; 2018.

© 2026 Chen E et al. *JAMA Network Open*.

32. Brody GH, Yu T, Miller GE, Chen E. Resilience in adolescence, health, and psychosocial outcomes. *Pediatrics*. 2016;138 e20161042.

33. Goodman E, Slap GB, Huang B. The public health impact of socioeconomic status on adolescent depression and obesity. *American Journal of Public Health*. 2003;93 1844-1850.

34. Nakamura JS, Wilkinson R, Nelson MA, Suzuki E, VanderWeele TJ. Volunteering in young adulthood: Complex associations with later health and well-being outcomes. *American Journal of Health Promotion*. 2025;39 39-51.

35. Hill CV, Perez-Stable EJ, Anderson NA, Bernard MA. The National Institute of Aging health disparities research framework. *Ethnicity and Disease*. 2015;25 245-254.

36. Gudi-Mindermann H, White M, Roczen J, Riedel N, Dreger S, Bolte G. Integrating the social environment with an equity perspective into the exposome paradigm: A new conceptual framework of the social exposome. *Environmental Research*. 2023;233 116485.

37. Senier L, Brown P, Shostak S, Hanna B. The socio-exposome: Advancing exposure science and environmental justice in a post-genomic era. *Environmental Sociology*. 2017;3 107-121.

© 2026 Chen E et al. *JAMA Network Open*.
