# Supplementary material for: Volunteering and Metabolic Syndrome and Diabetes in Black Adolescents From Low-Income Families
Source: JAMA Netw Open. 2026 Jan 12;9(1):e2553419. doi: 10.1001/jamanetworkopen.2025.53419 (PMC12797099; doi:10.1001/jamanetworkopen.2025.53419)
Supplement: Supplement 2. — Data Sharing Statement [file jamanetwopen-e2553419-s002.pdf]

Data Sharing Statement

Chen. Volunteering and Metabolic Syndrome and Diabetes in Black Adolescents From Low-Income Families. *JAMA Netw Open*. Published January 12, 2026.
doi:10.1001/jamanetworkopen.2025.53419

Data
Data available: No
